# Contrast-enhanced ultrasound for the differential diagnosis of thyroid nodules: An updated meta-analysis with comprehensive heterogeneity analysis

**DOI:** 10.1371/journal.pone.0231775

**Published:** 2020-04-20

**Authors:** Juanjuan Zhang, Xiuting Zhang, Yanna Meng, Yinghong Chen

**Affiliations:** 1 Department of Ultrasound, Huaihe Hospital of Henan University, Henan, China; 2 Department of Ultrasound, First Affiliated Hospital of Zhengzhou University, Zhengzhou, China; Kaplan Medical Center, ISRAEL

## Abstract

The diagnostic accuracy of contrast-enhanced ultrasound (CEUS) for distinguishing malignant thyroid nodules from benign thyroid nodules remains controversial. This meta-analysis was performed to evaluate the overall diagnostic value of CEUS for the characterization of thyroid nodules. Relevant studies were identified by searching PubMed, Embase and the Cochrane Library until August 1^th^ 2019 to assess the overall diagnostic accuracy of CEUS. 37 eligible studies were included in the present meta-analysis. The pooled sensitivity, specificity, positive likelihood rate, negative likelihood rate and diagnostic odds ratio of CEUS were 0.87, 0.83, 5.38, 0.17 and 38.94, respectively, with the AUC of 0.9263. Subgroup analysis showed the heterogeneity was greatly reduced in small nodules group (≤ 1 cm) (*I*^*2*^ *=* 0.0%), while heterogeneity was still observed in the group of variable sizes group (*I*^*2*^ = 69.5%). However, meta-regression analysis revealed that only diagnostic criterion was the major source of heterogeneity (*p* = 0.0259). The risk of publication bias was negligible (*p* = 0.35). CEUS exhibited high accuracy for the identification of thyroid nodules and might provide additional perfusion information for the current US imaging reporting systems.

## Introduction

A thyroid nodule, by definition, is a discrete lesion within the thyroid gland, which is radiographically distinct from the surrounding thyroid parenchyma [1]. With the increased utilization of diagnostic imaging (ultrasound (US), computed tomography (CT) or magnetic resonance imaging (MRI)) unrelated to the thyroid, the prevalence of thyroid incidentaloma has been significantly increased in the past twenty years [2,3]. For instance, the proportion of patients with thyroid nodules that clinicians now encounter is as high as 68% of the general population [4]. The major clinical issue in managing thyroid nodules is to maximize the detection of relevant thyroid cancer while reducing unnecessary overdiagnosis and overtreatment [5,6].

Several diagnostic tools have been used to identify nodules. Fine needle aspiration biopsy (FNAB) is widely adopted by clinicians as a simple way of diagnosing thyroid nodules [2]. However, this invasive technique may have false positive or negative outcomes, and about 10% to 20% of thyroid nodules could not be diagnosed [7]. Conventional US is the first-line diagnostic tool for initial cancer risk stratification of thyroid nodules [2,7]. Certain ultrasound features associated with malignancy include solid composition, hypoechogenicity, infiltrative or irregular margins, intranodular blood flow, microcalcification and “taller-than-wide” configuration [7,8]. However, one single ultrasound characteristic has inadequate sensitivity or specificity to identify or rule out malignant tumors. For example, microcalcification has the highest positive predictive value (PPV) among the above ultrasound features, but it has low sensitivity and only exists in 26% to 59% thyroid cancers [9]. In order to increase diagnosis confidence, the American Thyroid Association (ATA), American College of Radiologists (ACR) and other professional groups have proposed several systems to classify nodules by sonographic features and to recommend cutoffs for FNAB [2]. Among them, the thyroid imaging reporting and data system (TI-RADS) is a simple and practical reporting system created by ACR [10]. Horvath [11] and Kwak [12] indicated TI-RADS could be used to improve diagnostic accuracy, reduce unnecessary biopsies and follow-up scans, and ultimately improve management of patients with thyroid nodules. Despite TI-RADS has achieved great clinical value, it still suffers inherent flaws, such as dependence on experience, poor repeatability and high heterogeneity among sonographers [7].

**Fig 1:**
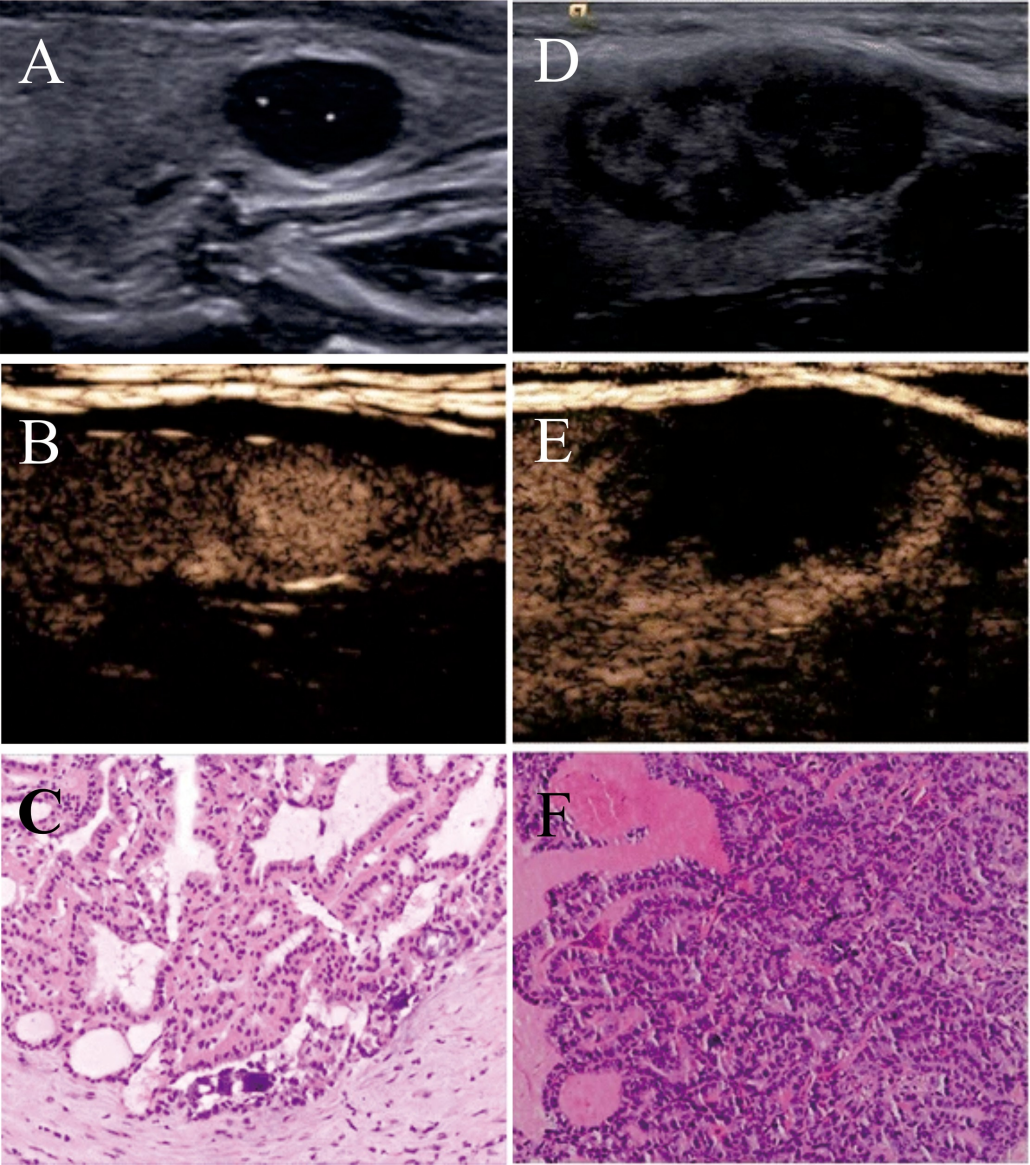
(**A**~**C**) A 35-year -old man was found to have a solid nodule in the left lobe of his thyroid. (**A**) The conventional two-dimensional image showed the nodule had three malignant indicators (solid, markedly hypoechoic, and microcalcifications). (**B**) CEUS image showed high enhancement of the nodule, indicating that the nodule was benign. (**C**) The pathological result revealed this lesion was a nodular goiter. (**D**~**F**) A 42-year-old woman was found to have a solid nodule in her left thyroid lobe. (**D**) The conventional two-dimensional image showed the nodule had two malignant indicators (solid and irregular margin). (**E**) CEUS image showed low enhancement of the nodule, indicating that the nodule was malignant. (**F**) The pathological result revealed this lesion was a thyroid papillary carcinoma [15]. From Zhang et al. (2017) Usefulness of combined use of contrast-enhanced ultrasound and TI-RADS classification for the differentiation of benign from malignant lesions of thyroid nodules. Eur Radiol 27: 1527–1536. doi: 10.1007/s00330-016-4508-y.

Advances in US technology may facilitate better characterization of benign and malignant thyroid nodules. Contrast-enhanced ultrasound (CEUS) is currently the focus of medical US research, because it can show microvascular blood flow clearly and assess tumor perfusion and vascular distribution in real time after intravenous injection of microbubble contrast agent (CA) [13]. It is well known that the thyroid gland is rich in microvessels, thus the parenchyma of normal thyroid exhibits rapid uniform enhancement after injection of CA [14]. However, the vascular structures of nodules differ from normal tissues, which enhance their differences from normal tissues [14]. Several studies [15,16] revealed specific CEUS enhancement patterns (such as heterogeneous or low enhancement) were related to malignant thyroid nodules (Fig 1). Unfortunately, not all studies confirmed these results [17]. Furthermore, CEUS presents some limitations: the examination is expensive, only one nodule can be evaluated for each injection of CA, and there was no established criterion for the patterns of enhancement and classification of thyroid nodules, resulting in the inability to be widely-used worldwide [14]. Besides, the current thyroid nodule imaging reporting systems, including the widely-used TI-RADS, do not contain CEUS enhancement patterns as the evaluation item [2], making it controversial in clinical practice. Therefore, the present comprehensive meta-analysis was conducted to investigate the value of CEUS for distinguishing malignant thyroid nodules from benign ones, hoping to provide evidence for the new TI-RADS classification system including CEUS features.

## Methods

This present meta-analysis was conducted following the PRISMA statement. No institutional review board approval is required, as our study was a systematic review and meta-analysis, thus obtaining consent was exempted for this study.

### Literature search

A comprehensive search was undertaken to identify suitable articles from electronic databases (the PubMed, Embase, and Cochrane Library) without an upper-limit date until August 1^th^, 2019. Searches were performed using the following MeSH heading and key words: “thyroid” and “CEUS or contrast-enhanced ultrasound or contrast-enhanced US or contrast-enhance Doppler ultrasonography or contrast-enhanced ultrasonography”. Additionally, manual retrieval was also performed on references of related reviews.

### Eligibility and exclusion criteria

After removing duplicate studies, two authors independently reviewed the titles and abstracts based on the following inclusion and exclusion criteria. (A) inclusion criteria: (1) related to the diagnostic value of CEUS for thyroid nodules; (2) all patients were diagnosed with histopathology as the gold standard; (3) reported data were sufficient to calculate the true-positive (TP), false-negative (FN), false-positive (FP) and true-negative (TN). (B) exclusion criteria: (1) studies not published in English; (2) duplicated publications or incomplete data; (3) reviews, letters, case reports and editorials; (4) studies not performed on human subjects. When two authors’ opinions differed, the disagreements would be resolved by consensus.

### Data extraction and quality evaluation

Full texts of eligible studies were screened and data were extracted using a standardized form for the final meta-analysis. The detailed information including first author, publication year, country, specimen, sample size (both lesions and patients), gold standard, diagnostic criterion, type and dosage of contrast agent, TP, FP, TN, FN and any other additional information required for quality evaluation.

The methodological quality of eligible studies was assessed by the Quality Assessment of Diagnostic Accuracy Studies (QUADAS) criteria, which are recommended for diagnostic systematic reviews based on sources of bias and variation.

### Statistical analysis

The statistical softwares Meta-Disc (version 1.4, XI Cochrane Colloquium, Barcelona, Spain) and STATA (version 11.0, Stata Corporation, College Station, TX, USA) were used in this meta-analysis. The pooled sensitivity, specificity, positive likelihood ratio (PLR), negative likelihood ratio (NLR), diagnostic odds ratio (DOR), and their corresponding 95% confidence intervals (CI) were calculated by certain formulae composed of TP, FP, TN and FN. Besides, the summary receiver operating characteristic (SROC) curve and areas under curve (AUC) were also constructed. The heterogeneity of included studies was investigated using *I*^*2*^ and *Q* tests. The random-effects model (DerSimonian-Laird method) was used because the heterogeneity was significant in this meta-analysis. Spearman correlation coefficient was calculated to analyze the diagnostic threshold. Subgroup analysis and meta-regression analysis were subsequently performed to assess the variabilities attributed to heterogeneity. Publication bias was investigated by Deeks’ funnel plot analysis. Two-sided statistical tests were performed, with a *p* value < 0.05 representing statistically significant.

## Results

### Study selection

**Fig 2:**
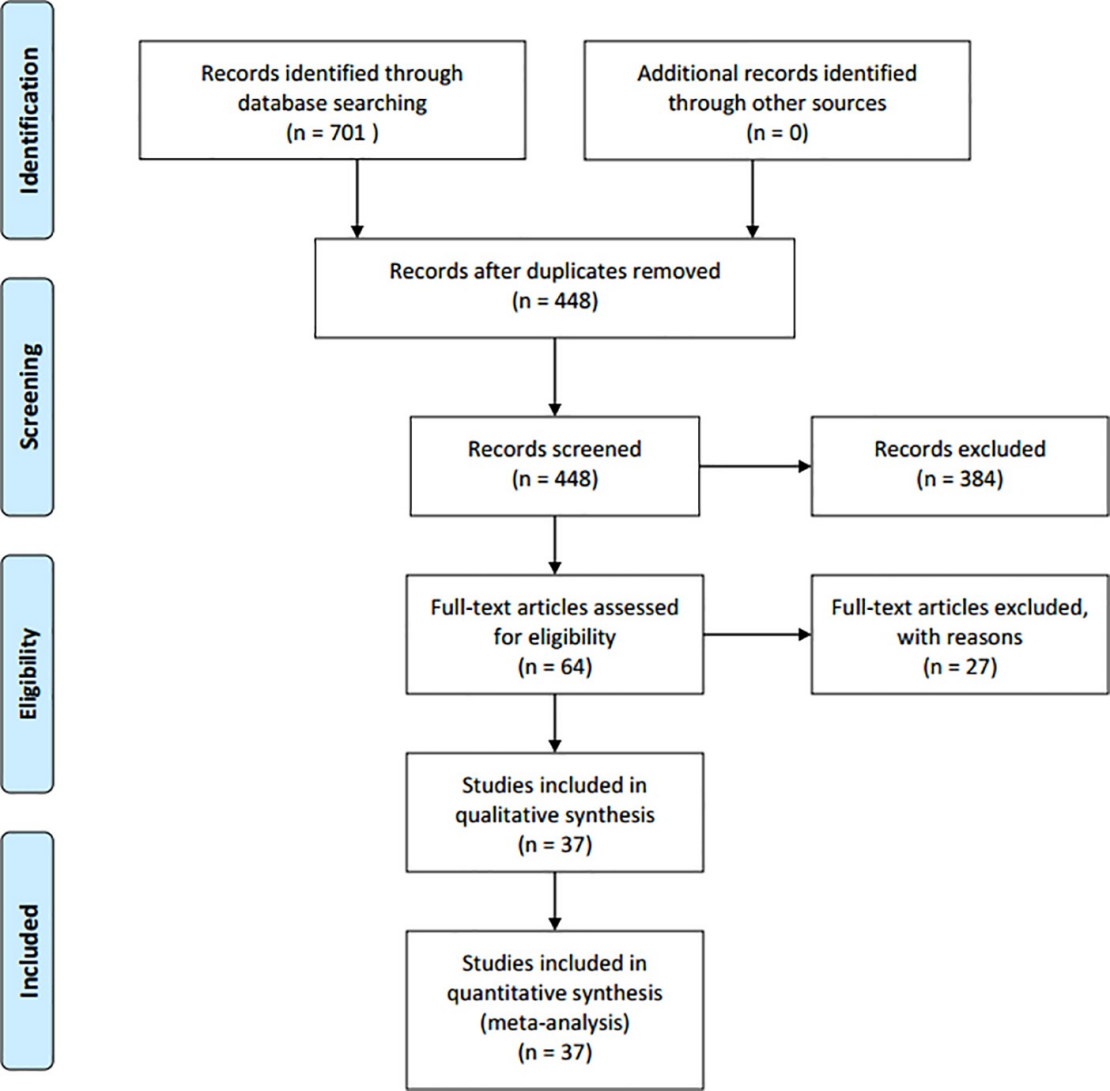
Process of literature search and selection.

Initial electronic search identified 701 records, 311 from PubMed, 381 from Embase and 9 from Cochrane. After automatically and manually removing the duplicates, 448 studies were left for initial screening. Depending on the inclusion and exclusion criteria, 384 studies were excluded. Full texts of the remaining 64 studies were left for further reviewing. Finally, 37 [14–16, 18–51] studies were identified as eligible studies. The search and selection process for this meta-analysis was shown in Fig 2.

### Characteristics and quality assessment of the included studies

The basic characteristics of the included 37 studies were summarized in Table 1. The publication year of studies ranged from 2010 to 2019. These studies were from 4 countries, most of them (29 studies) were done in Asia (all from China), the rest 8 were from Europe (5 from Italy, 2 from Germany and 1 from Austria). The average age of all included patients was under 60. The number of lesions varied from 20 to 432, and the number of patients varied from 20 to 370. Most of the malignant lesions were papillary carcinomas, and most of the benign lesions were nodular goiters and adenomas. Most of contrast agents used in the studies were Sonovue, except for two studies that did not mention the specific brand. The dosages of contrast agent ranged from 1 to 5 mL. QUADAS scores were also summarized in Table 1.

**Table 1:** Basic characteristics and quality assessment of the eligible studies included in this meta-analysis.

	First author	Year	Country	Average age	Male / Female	Number of lesions	Number of patients	Average tumor size (mm)	Contrast agent (type and dosage)	TP	TN	FP	FN	QUADAS score
1	Zhao [18]	2019	China	46	NA	117	108	14	Sonovue, 2.4 mL	51	53	7	6	12
2	Xu [19]	2019	China	43	68/302	432	370	NA	Sonovue, 5 mL	221	145	29	37	12
3	He [20]	2018	China	46	NA	88	83	29	Sonovue, 1.8 mL	23	54	5	6	11
4	Tian [21]	2018	China	43	55/69	162	124	NA	Sonovue, 1.5 mL	50	95	9	8	12
5	Wang [22]	2018	China	49	32/103	135	135	12	Sonovue, 1.8 mL	47	64	24	0	10
6	Zhang [23]	2018	China	43	NA	120	NA	19	Sonovue, 2 mL	41	77	1	1	10
7	Zhao [24]	2018	China	46	84/223	367	307	NA	Sonovue, 1.2 mL	178	106	44	39	11
8	Caresio [25]	2018	Italy	M(43); F(46)	3/17	20	20	Malignant(26); benign(20)	Sonovue, 2.4 mL	10	10	0	0	9
9	Wang [16]	2018	China	Malignant(48); benign(50)	32/103	135	135	Malignant(12); benign(13)	NA, 1.8mL	47	64	24	0	11
10	Zhou [26]	2018	China	44	43/118	167	161	13	Sonovue, 2.4 mL	78	56	14	19	10
11	Jin [27]	2017	China	44	19/58	77	74	Malignant(11); benign(13)	Sonovue, 1.5–2.5 mL	26	34	7	10	10
12	Zhang [15]	2017	China	46	85/161	319	246	12	Sonovue, 2.4 mL	58	230	14	17	11
13	Diao [28]	2017	China	52	NA	87	77	14	Sonovue, 1.5 mL	51	26	6	4	11
14	Li [29]	2017	China	43	21/68	89	89	13	Sonovue, 2.4 mL	52	29	4	4	12
15	Liu [30]	2017	China	40	33/67	122	100	NA	NA, 1.6mL	51	60	5	6	9
16	Ma [31]	2017	China	49	NA	135	125	≤ 10	Sonovue, 2.4 mL	66	47	9	13	9
17	Chen [32]	2016	China	NA	NA	319	253	NA	Sonovue, 1.2 mL	119	158	25	17	10
18	Zhang [33]	2016	China	45	NA	157	148	12	Sonovue, 2.4 mL	81	32	43	1	11
19	Sui [34]	2016	China	49	50/47	109	97	Malignant(24); benign(23)	Sonovue, 2.4 mL	54	39	4	12	12
20	Wendl [35]	2016	Germany	52	17/33	50	50	NA	Sonovue, 2.4 mL	16	19	11	4	10
21	Wu [36]	2016	China	NA	NA	133	NA	NA	Sonovue, 1.2 mL	81	32	16	4	9
22	Zhang [37]	2016	China	Malignant(42); benign(54)	20/91	145	111	Malignant(13); benign(17)	Sonovue, 1.3 mL	54	56	26	9	9
23	Jiang [38]	2015	China	46	37/85	122	122	15	Sonovue, 2.4 mL	44	67	6	5	11
24	Li [39]	2015	China	40	21/52	80	73	NA	Sonovue, 2.4 mL	44	24	6	6	10
25	Yuan [40]	2015	China	40	32/46	78	78	18	Sonovue, 2.5 mL	35	36	5	2	10
26	Schleder [41]	2015	Germany	54	45/55	101	101	Malignant(32); benign(27)	Sonovue, 1–2.4 mL	24	61	14	2	9
27	Zhao [42]	2015	China	46	22/61	102	83	Malignant(7); benign(7)	Sonovue, 1.2 mL	51	32	7	12	10
28	Liang [43]	2014	China	30	45/35	80	80	19	Sonovue, 2.4 mL	22	46	12	0	9
29	Giusti [44]	2014	Italy	NA	NA	53	NA	NA	Sonovue, 4.8 mL	7	30	10	6	11
30	Jiang [45]	2014	China	45	37/85	122	122	Malignant(8); benign(20)	Sonovue, 2.4 mL	60	57	3	2	9
31	Deng [46]	2014	China	46	42/104	175	146	18	Sonovue, 2.4 mL	46	101	18	10	10
32	Ma [47]	2014	China	48	39/105	172	144	NA	Sonovue, 1.5 mL	85	71	7	9	11
33	Cantisani [48]	2013	Italy	49	13/35	53	48	20	Sonovue, 4.8 mL	15	31	3	4	9
34	Giusti [14]	2013	Italy	NA	NA	38	33	NA	Sonovue, 4.8 mL	15	11	5	7	8
35	Acharya [49]	2012	Italy	M(54); F(50)	10/10	20	20	NA	Sonovue, 2.5 mL	10	10	0	0	10
36	Nemec [50]	2012	Austria	NA	NA	42	42	NA	Sonovue, 2.4 mL	8	26	5	3	9
37	Zhang [51]	2010	China	M(49); F(43)	21/74	104	95	Malignant(26); benign(25)	Sonovue, 1.6 mL	45	49	4	6	10

M male, F female, NA not available, QUADAS quality assessment tool for diagnostic accuracy studies, TP true-positive, TN true-negative, FN false-negative, FP false-positive.

### The overall diagnostic accuracy

**Fig 3:**
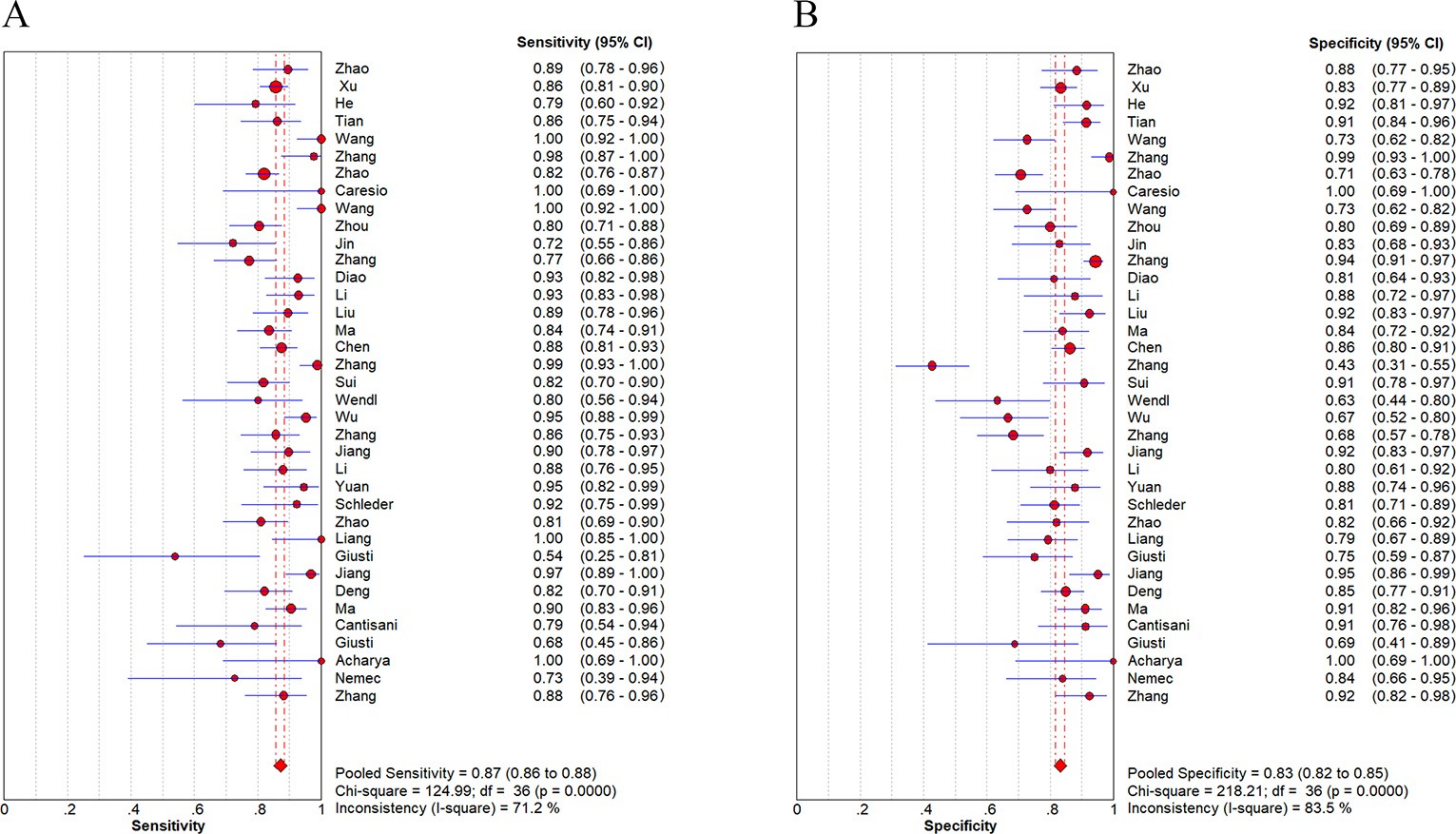
Sensitivity (**A**) and specificity (**B**) of CEUS for characterizing thyroid nodules.

**Fig 4:**
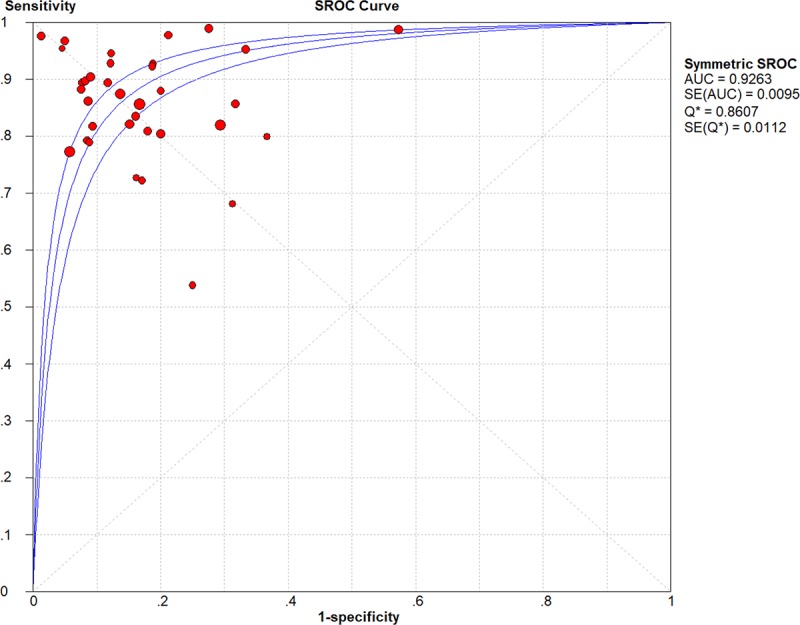
SROC curves of CEUS for characterizing thyroid nodules.

Analysis of CEUS for characterizing thyroid nodules showed that the pooled sensitivity was 0.87 (95%CI: 0.86–0.88), and specificity was 0.83 (95%CI: 0.82–0.85) (Fig 3). The pooled PLR, NLR and DOR were 5.38 (95%CI: 4.28–6.76) and 0.17 (95%CI: 0.14–0.20), 38.94 (95%CI: 27.69–54.75), respectively (S1 Fig). Fig 4 illustrated the SROC curve with AUC to be 0.9263, which was close to 1, indicating CEUS as a useful diagnostic tool to distinguish malignant from benign thyroid nodules. As seen in the forest plots (Fig 3 and S1 Fig), all indices of diagnostic accuracy denoted heterogeneity of the included studies. There was no threshold effect by calculating spearman correlation coefficient (*r* = -0.093, *p* = 0.585).

### Subgroup analysis and meta-regression analysis

**Fig 5:**
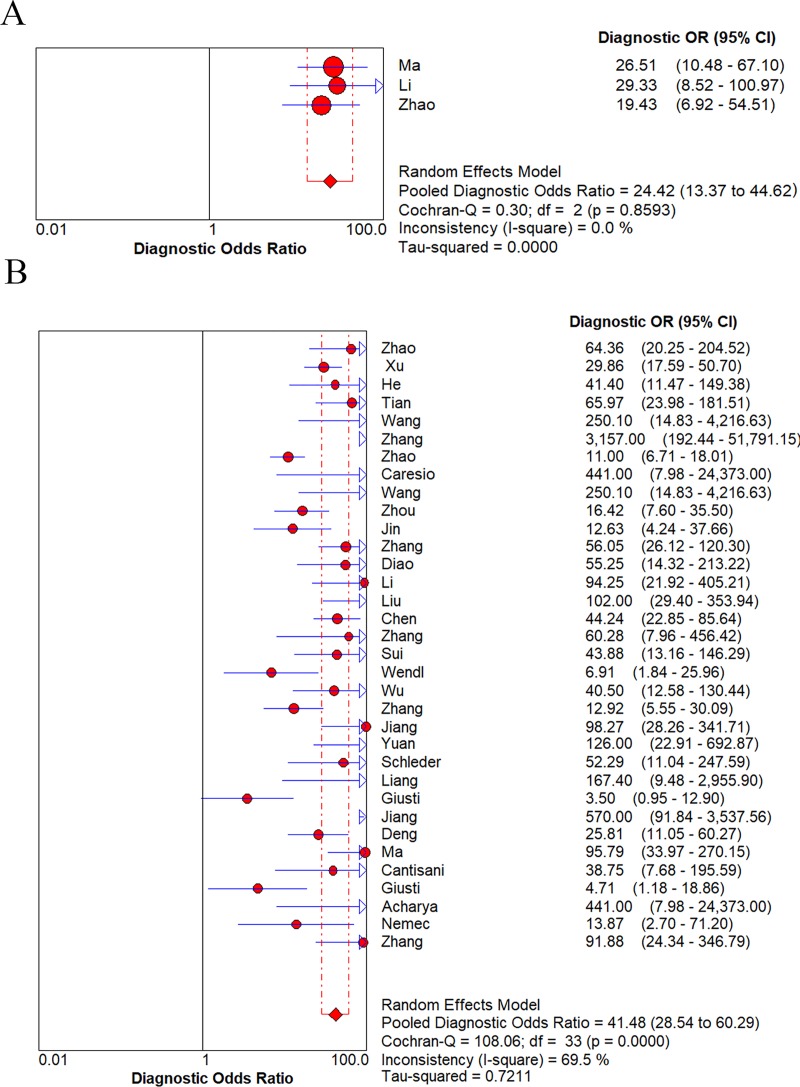
Subgroup analysis of DOR for small nodules (**A**) and variable sizes of nodules (**B**) in characterization of thyroid nodules.

Because the DOR is a single estimate which combines the data from sensitivity and specificity, the pooled DOR was calculated to present the diagnostic accuracy. Several potential factors were explored to show their abilities in affecting the diagnostic accuracy. As the results listed in Table 2, region, number of lesions, size of nodules and CA dosage greatly influenced the diagnostic accuracy. DOR of Asian country (all from China) was improved than it from Western countries (45.85 vs. 16.31). Similarly, the higher the number of lesions, the higher the diagnostic efficiency (46.52 vs. 26.65). And DOR of low CA dosage group was higher than them of high CA dosage group and variable dosage group (50.96 vs. 34.71 vs. 34.71). However, great heterogeneity was observed in all above groups (*I*^*2*^ all *>* 50%). Besides, size of nodules also greatly affected the diagnostic accuracy of included studies (Fig 5). DOR of variable sizes group was higher than small nodules group (41.48 vs. 21.42). However, the heterogeneity was greatly reduced in small nodules group (≤ 1 cm) (*I*^*2*^ *=* 0.0%), while great heterogeneity was observed in the group of variable sizes of nodules (*I*^*2*^ = 69.5%). Diagnostic accuracy was equivalent according to different diagnostic criterion. When Sonographers characterized the thyroid nodules by visual features (such as heterogeneous enhancement was identified as malignancy), its overall diagnostic accuracy was the same as when using quantitative or semi-quantitative parameters (both DOR were 53.46). However, great heterogeneity was observed in both above groups (both *I*^*2*^ were 64.4%).

**Table 2:** Subgroup analysis of DOR of CEUS for the diagnostic performance of thyroid nodules.

Subgroup	Number of studies	Pooled DOR	95%CIs	*I*^*2*^	*p* value
**Region**					
Western countries	8	16.31	6.09–43.71	60.5%	0.0133
Asian country	29	45.85	32.38–64.92	65.3%	< 0.0001
**Number of lesions**					
≤ 100	14	26.65	13.37–53.10	61.9%	0.0011
> 100	23	46.52	31.41–68.92	69.4%	< 0.0001
**Size of nodules**					
Small nodules (≤ 1 cm)	3	24.42	13.37–44.62	0.0%	0.8593
Variable sizes of nodules	34	41.48	28.54–60.29	69.5%	< 0.0001
**CA dosage**					
≤ 2 mL/person	14	50.96	28.05–92.58	75.1%	< 0.0001
> 2 mL/person	21	34.71	20.07–54.58	62.9%	0.0001
Variable dosages	2	34.71	22.07–54.58	62.9%	0.0001
**Diagnostic criterion**					
Visual features	24	53.46	36.34–78.64	64.4%	< 0.0001
Quantitative or semi-quantitative parameters	13	53.46	36.34–78.64	64.4%	< 0.0001

DOR diagnostic odds ratio, CIs confidence intervals, CA contrast agent.

Meta-regression analysis was also performed to take all the above factors into account. As seen in Table 3, diagnostic criterion was the major source of heterogeneity (*p* = 0.0259). However, size of nodules was not the source of heterogeneity (*p* = 0.1084).

**Table 3:** Meta-regression analysis of potential sources of heterogeneity.

Potential sources	Coeff	Std. Err	*p* value	RDOR	UL	LL
**Region**	0.599	0.5756	0.3067	1.82	0.56	5.89
**Number of lesions**	-0.046	0.4163	0.9127	0.96	0.41	2.23
**Size of nodules**	-0.911	0.5505	0.1084	0.40	0.13	1.24
**CA dosage**	-0.042	0.3002	0.8906	0.96	0.52	1.77
**Diagnostic criterion**	-0.972	0.4148	0.0259	0.38	0.16	0.88

RDOR relative diagnostic odds ratio, UL upper limit, LL lower limit, CA contrast agent.

### Publication bias

**Fig 6:**
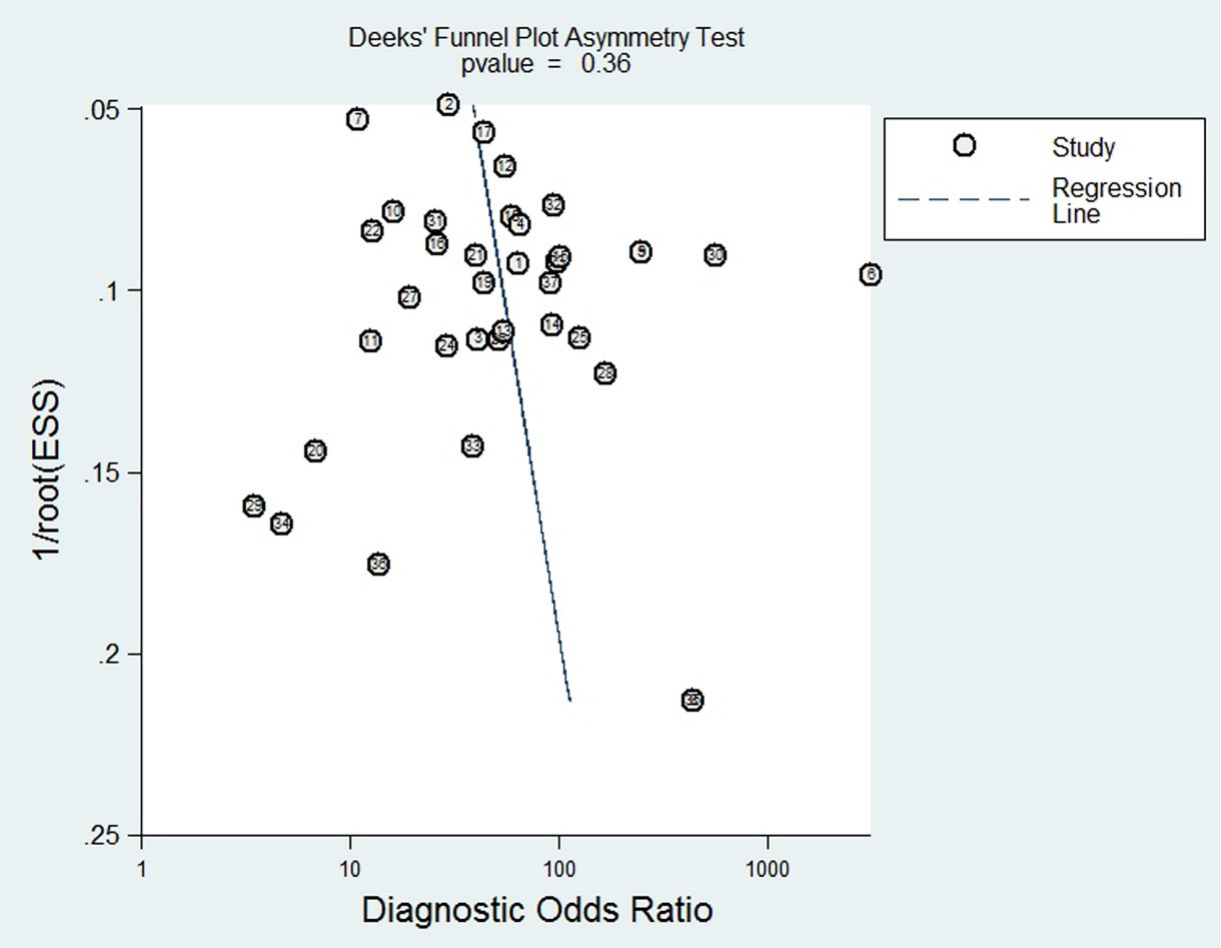
Funnel plot for the evaluation of potential publication bias.

A Deeks’ funnel plot was generated to assess the publication bias of the 37 eligible studies. As seen in Fig 6, the plot was symmetric, indicating that there was no potential publication bias for the included studies (*p* = 0.35).

## Discussion

### The diagnostic accuracy of CEUS

Early diagnosis and correct identification of thyroid cancer has great importance for clinical treatment regimens and may improve the prognosis [32]. However, differentiating malignancy from benign thyroid nodules remains the challenging dilemma. The combination of several suspicious US characteristics can further improve the diagnostic accuracy than one single US characteristic. A large number of studies have used TI-RADS system to predict malignant thyroid nodules [12,13]. But this system is still limited to different US criterion and inevitable inter-observer variability, making its sensitivity and specificity compromised. CEUS promises to bring additional diagnostic power to this conventional imaging modality with its high sensitivity for detecting the microvascularization of thyroid carcinomas [15]. Zhang [15] and Zhao [18] implied CEUS had complementary effects to the TI-RADS system, which improved the differential diagnosis of thyroid nodules. Jiang [38] also demonstrated CEUS could even provide a basis for determining which nodules should undergo fine needle aspiration (FNA). Whereas, Friedrich-Rust [52] indicated that CEUS did not improve the characterization of thyroid nodules in their preliminary study. Because of these conflicting studies, clinicians are often confused when they choose imaging techniques. Therefore, a comprehensive meta-analysis is in an urgent need to summarize all current results to determine the role of CEUS for characterizing thyroid nodules. Despite Liu et al [53] (search date was May 2018) and Ma et al [54] (search date was December 2013) had published their meta-analyses on this topic, we noticed there were repeated studies included in both researches, which compromised the diagnostic performance. Additionally, significant heterogeneity existed in both studies, but neither of them pursued its source. However, heterogeneity analysis is very important, it is an essential part of systematic review, and it can provide a basis for assessment of diagnostic research. For these reasons, an updated meta-analysis was performed, including more preliminary studies and comprehensive heterogeneity analysis to assess the performance of CEUS in identifying benign and malignant thyroid nodules.

Our results showed that CEUS had high pooled sensitivity and specificity (0.87 and 0.83) with AUC of 0.9263, indicating it could be a considerable tool in the diagnosis of thyroid nodules. Moreover, funnel plot analysis revealed absence of publication bias, which strengthened the validity of the study. However, there was great heterogeneity of included studies, which might compromise the credibility. Thus, subgroup and meta-regression analyses were conducted to reveal potential sources of heterogeneity.

### Heterogeneity analysis of this meta-analysis

Subgroup analysis showed great heterogeneity in the variable-sized nodule group (*I*^*2*^ = 69.5%), while the small nodule group (≤1 cm) was significantly reduced (*I*^*2*^ = 0.0%). Unfortunately, meta-regression analysis did not show that nodular size was a source of heterogeneity. Several studies reported that CEUS features were closely related to nodule size [31, 39]. Yuan et al [39] implied in thyroid cancers less than 1 cm in diameter, the hypoenhancement was more common than thyroid cancers with diameters greater than 2 cm. Ma’s [31] research showed that heterogeneous enhancement was an independent predictor for predicting papillary thyroid microcarcinoma (PTMC). In all eligible studies, there were three studies [31, 39, 42] focused on the diagnosis of PTMC from small nodules (≤ 1 cm). Among them, two studies [31, 42] used heterogeneous enhancement as the diagnostic criterion for PTMC, the remaining one [39] considered inhomogeneous perfusion and whole course low enhancement as the diagnostic indicators for PTMC. Due to similar diagnostic criterion, the heterogeneity was greatly reduced in this group (*I*^*2*^ *=* 0.0%), while great heterogeneity was still observed in the group of variable sizes of nodules (*I*^*2*^ = 64.5%). However, Li [39] demonstrated that CEUS had no significant advantage in characterizing PTMC for the following reasons: firstly, there were overlapped CEUS perfusion features in small malignant and benign nodules; secondly, small malignant tumors might not exhibit typical characteristics of malignancies; thirdly, the instrument sensitivity, adjustments and parameters employed during the process of imaging might fail to meet diagnostic level at present. Similar to the above hypotheses, our subgroup analysis showed relatively lower diagnostic accuracy for small nodules group than variable sizes group (DOR: 24.42 vs. 41.48).

Meta-regression analysis revealed diagnostic criterion was the major factor which contributed to the great heterogeneity. All diagnostic criteria of included studies could be divided into two categories: visual features and quantitative or semi-quantitative parameters. Most of the studies (24 studies) used visual features (perfusion characteristics observed by sonographer observed during the procedure) to differentiate thyroid nodules, which were usually low or weak enhancement pattern, heterogeneous enhancement pattern and ill-defined enhancement border. Because papillary thyroid cancers (PTC) account for the vast majority of thyroid cancers, their characteristics contributed to the main diagnostic criterion. According to these studies, most thyroid cancers showed hypoenhancement due to insufficient blood supply in PTC [8]. The necrosis inside the cancer, blocked blood vessels by cancer embolus, intensive interstitial fibrosis and thyroid peripheral calcification may all lead to the hypoenhancement feature of CEUS in thyroid cancers [27]. While benign nodules (such as nodular goiters and adenomas) usually have enhanced perfusion, homogeneous enhancement and no perfusion defects because of their rich blood supply [30]. Our meta-analysis results showed visual features had favorable ability to identify malignant nodules with DOR of 53.46, however, the heterogeneity of this group was still considerable (*I*^*2*^ *=* 64.4%). With the introduction of ultrasound software and computer-aided diagnosis (CAD) technology, quantitative or semi-quantitative parameters derived from series of CEUS images are believed to provide more valuable information for blood flow observation than visualization [27]. Commonly used parameters are derived from the time-intensity curve. Zhou [26] showed the nodule-to-perinodule peak intensity ratio had the best diagnostic efficiency for identifying malignant thyroid nodules. Jin [27] utilized the image heterogeneity calculation equation to calculate the heterogeneity values (HVs) and heterogeneity ratios (HRs), which were quantifications of nodular heterogeneity enhancement. And their results revealed the heterogeneity quantifications had lower sensitivity (0.72 vs. 0.83), but higher specificity (0.83 vs. 0.66) and diagnostic accuracy (0.78 vs. 0.74) than visual assessment. Whereas, the parameters used in their studies were quite different from one by one, which might lead to great heterogeneity (*I*^*2*^ *=* 64.4%) in this group (quantitative or semi-quantitative parameters). Therefore, diagnostic criterion would need further investigation and standardization. Especially, standardized diagnostic parameters need to be determined and validated in the future.

## Conclusion

In conclusion, CEUS might be a promising method for identifying the malignancy from benign thyroid nodules. Yet, there is still insufficient evidence that CEUS features can improve the diagnostic accuracy of US imaging reporting systems (such as TI-RADS) at present, our meta-analysis provided the potential for forward-looking, multi-center and large-scale CEUS studies in the future.

## Supporting information

S1 Checklist PRISMA 2009 checklist. (DOC)

S1 Fig PLR (**A**), NLR (**B**) and DOR (**C**) of CEUS for characterizing thyroid nodules.
(TIF)
