# Supplementary figures and images for: Contrast-enhanced ultrasound for the differential diagnosis of thyroid nodules: An updated meta-analysis with comprehensive heterogeneity analysis
Source: PLoS One. 2020 Apr 20;15(4):e0231775. doi: 10.1371/journal.pone.0231775 (PMC7170259; doi:10.1371/journal.pone.0231775)

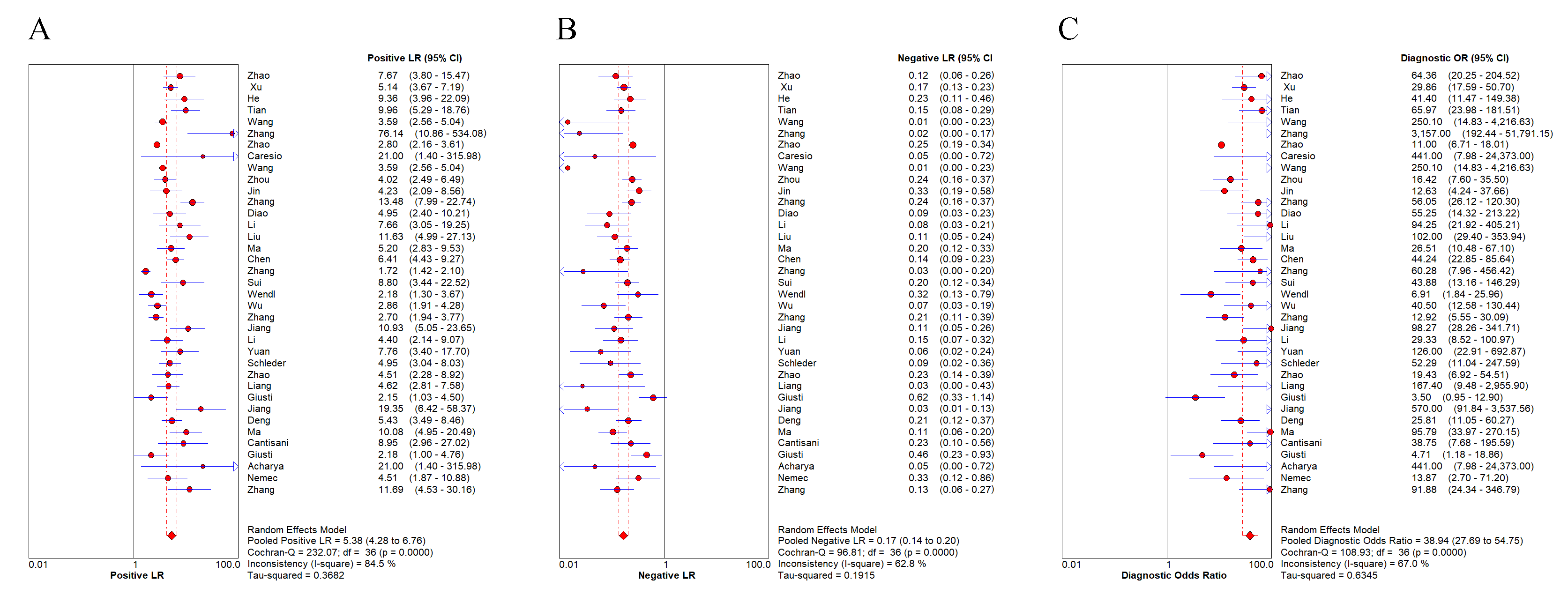

Supplement: S1 Fig — PLR (A), NLR (B) and DOR (C) of CEUS for characterizing thyroid nodules. (TIF) [file pone.0231775.s002.tif]
